# Diagnosis and management of temperature abnormality in ICUs: a EUROBACT investigators' survey

**DOI:** 10.1186/cc13153

**Published:** 2013-12-10

**Authors:** Daniel J Niven, Kevin B Laupland, Alexis Tabah, Aurélien Vesin, Jordi Rello, Despoina Koulenti, George Dimopoulos, Jan de Waele, Jean-Francois Timsit

**Affiliations:** 1 Critical Care Medicine, Peter Lougheed Centre and University of Calgary, 3500 26th Street NE, Calgary, AB T1Y 6J4, Canada; 2 Intensive Care Unit, Royal Inland Hospital, 311 Columbia Street, Kamloops, BC V2C 2T1, Canada; 3 Albert Bonniot Institute, Team 11: Outcome of Mechanically Ventilated Patients and Respiratory Cancers, Université Grenoble 1, U 823, Rond-point de la Chantourne, Grenoble 38042, France; 4 Department of Intensive Care Medicine, Royal Brisbane and Women's Hospital, Butterfield Street, Brisbane, QLD 4029, Australia; 5 Burns, Trauma, and Critical Care Research Centre, The University of Queensland, Butterfield Street, Brisbane, QLD 4029, Australia; 6 Critical Care Department, Vall d'Hebron University Hospital, Passeig Vall d"Hebron, Barcelona 08035, Spain; 7 CIBERES, Universitat Autonoma de Barcelona, Barcelona 08035, Spain; 8 Department of Critical Care, University Hospital ATTIKON, Medical School University of Athens, 1 Rimini Street, Athens 14569, Greece; 9 Ghent University Hospital, 185 De Pintelaan, Ghent 9000, Belgium; 10 Medical ICU, Albert Michallon University Hospital, Université Grenoble 1, Blvd de la Chantourne, Grenoble 38043, France

**Keywords:** Fever, hypothermia, intensive care unit, sepsis, septic shock, bacteremia

## Abstract

**Introduction:**

Although fever and hypothermia are common abnormal physical signs observed in patients admitted to intensive care units (ICU), little data exist on their optimal management. The objective of this study was to describe contemporary practices and determinants of management of temperature abnormalities among patients admitted to ICUs.

**Methods:**

Site leaders of the multi-national EUROBACT study were surveyed regarding diagnosis and management of temperature abnormalities among patients admitted to their ICUs.

**Results:**

Of the 162 ICUs originally included in EUROBACT, responses were received from 139 (86%) centers in 23 countries in Europe (117), South America (8), Asia (5), North America (4), Australia (3) and Africa (2). A total of 117 (84%) respondents reported use of a specific temperature threshold in their ICU to define fever. A total of 14 different discrete levels were reported with a median of 38.2°C (inter-quartile range, IQR, 38.0°C to 38.5°C). The use of thermometers was protocolized in 91 (65%) ICUs and a wide range of methods were reportedly used, with axillary, tympanic and urinary bladder sites as the most common as primary modalities. Only 31 (22%) of respondents indicated that there was a formal written protocol for temperature control among febrile patients in their ICUs. In most or all cases practice was to control temperature, to use acetaminophen, and to perform a full septic workup in febrile patients and that this was usually directed by physician order. While reported practice was to treat nearly all patients with neurological impairment and most patients with acute coronary syndromes and infections, severe sepsis and septic shock, this was not the case for most patients with liver failure and fever.

**Conclusions:**

A wide range of definitions and management practices were reported regarding temperature abnormalities in the critically ill. Documenting temperature abnormality management practices, including variability in clinical care, is important to inform planning of future studies designed to optimize infection and temperature management strategies in the critically ill.

## Introduction

Temperature abnormalities occur in approximately 50% of patients admitted to adult intensive care units (ICUs) and are associated with increased mortality in select groups of patients [1-8]. Although strong theoretical arguments exist both for and against the treatment of pyrexia, the current literature does not support an outcome benefit from a particular temperature control strategy in patients without acute neurological injury [9-15]. Those against fever control argue that pharmacologic anti-pyresis with non-steroidal anti-inflammatory drugs and/or acetaminophen is potentially toxic [16,17], and physical cooling may cause shivering which increases metabolic demand and patient discomfort. On the other hand, advocates for fever control argue for increased patient comfort and a decrease in the risk of multi-system organ failure. Hypothermia is associated with adverse outcome in patients admitted to ICUs and increases the risk for nosocomial infection [8,18].

A number of small clinical trials have investigated temperature control strategies in febrile critically ill patients [11-14,19]. Due to the limited guidance provided by these studies, temperature control in febrile patients remains inconsistent and further trials are needed. However, in order to inform the planning of future trials an appraisal of current attitudes and practices is needed. We therefore conducted a survey of investigators in the EUROBACT study [20], a large observational study investigating hospital acquired bacteremia in ICUs, in order to describe contemporary temperature treatment practices in an international context.

## Materials and methods

A survey exploring fever management practices was developed specifically for this study (Additional file 1). Questions in English surrounding treatment thresholds, temperature measurement modalities, treatment strategies, the presence of written treatment protocols, and treatment of selected patients were included. The survey was pre-tested by sequential administration to a number of non-participating unit directors and intensivists and was revised progressively for clarity. The survey was then administered to all site leads participating in the EUROBACT study [20] using a web-based form. Respondents were asked to answer based on the expected or average practice within their respective units. Initial non-respondents were sent email reminders to complete the survey at approximately two- and four-weeks post-initiation and after were contacted directly to request participation. Data on ICU characteristics were available for all units and was obtained from the unit-based survey component of the EUROBACT study. This study involved a voluntary survey of the EUROBACT study investigators. The EUROBACT study was approved by the Institutional Review Board at Paris-Saint Joseph and each participating center in EUROBACT complied with their local ethical institutional review board approval standards.

All analyses were conducted using Stata 11.2 (StataCorp, College Station, TX, USA). Analyses were primarily descriptive. Non-normally distributed (skewed) continuous data were reported as medians with interquartile ranges (IQR) and groups were compared using the Mann-Whitney test. Grouped categorical data were compared using the Fisher's exact test or chi^2 ^for multiple categories. A *P*-value less than 0.05 was considered to represent statistical significance.

## Results

Of the 162 ICUs originally included in EUROBACT, responses were received from 139 (86%) centers in 23 countries in Europe (117), South America (8), Asia (5), North America (4), Australia (3) and Africa (2). There were no statistically significant differences among responders and non-responders with regard to reported average ICU case-fatality rate, ICU specialty type, university-affiliation or public/private ICU. However, non-respondents had significantly larger ICUs (median 18 beds, IQR, 11 to 25 versus 12 beds, IQR, 9 to 18; *P *= 0.018) and were more likely to be from non-European centers (11/33; 33% vs 12/129; 9%; *P *= 0.001).

Among the 139 respondents, 117 (84%) reported use of a specific temperature threshold in their ICU to define fever. A total of 14 different discrete levels were reported to define fever ranging from 37.0°C to 40.0°C with a median of 38.2°C (IQR, 38.0°C to 38.5°C). The use of thermometers was protocolized in 91 (65%) ICUs. A wide range of temperature measurement methods were used, with axilla, pulmonary artery catheter, and rectal thermometry the most commonly reported modalities (Table 1). Axillary, tympanic and urinary bladder catheter thermometry were reported as the most common primary means of measurement (Table 1).

**Table 1:** Temperature measurement modalities used in EUROBACT intensive care units

Thermometer type/location	ICUs reporting usage	Primary measure
**Axillary**	102	58
**Pulmonary artery catheter**	78	1
**Rectal**	73	12
**Tympanic**	64	30
**PICCO**	59	2
**Esophageal**	51	4
**Urinary bladder**	50	16
**Temporal**	14	9
**Oral**	12	2
**Other**	8	1

When asked about new fever and the ordering of blood cultures, 83 (60%) reported these were only done by specific physician order, 25 (18%) stated these were routinely performed by nurses unless requested otherwise, 24 (17%) routinely performed these per protocol based on a predefined temperature threshold, and 7 (5%) indicated use of other approaches. When asked to report a usual threshold of hypothermia that would trigger the ordering of blood cultures, 129 (93%) responses were received. Fourteen different discrete levels were reported which ranged from 34°C to 38.3°C with a median of 36.0°C (IQR, 35.2°C to 36.0°C). In the event of hypothermia, 104 (75%) performed cultures only with specific physician order, 21 (15%) routinely cultured per protocol based on a specific temperature threshold, 10 (7%) routinely cultured blood as performed by nurses unless requested otherwise, and 4 (3%) indicated other approaches.

When asked specifically about ordering blood cultures in response to new fever or hypothermia, 136 (98%) indicated systematic use of aerobic culture bottles, 113 (81%) systematic use of anaerobic bottles, 37 (27%) systematic use of bottles for fungi, and 42 (30%) indicated that the type of bottles were specifically defined by physician order.

In the management of patients, only 31 (22%) respondents indicated that there was a formal written protocol in place for temperature control among febrile patients in their ICUs. In most or all cases the practice was to control temperature, to use acetaminophen and to perform a septic workup in febrile patients and that this was usually directed by physician order as shown in Table 2. While reported practice was to treat nearly all (median score 5 = always) patients with neurological impairment and most (median score 4 = most) patients with acute coronary syndromes and infections, severe sepsis and septic shock, this was not the case for many (median score 3 = sometimes) patients with liver failure as shown in Table 3. The responses for management (Tables 2 and 3) were similar among European and non-European respondents with two exceptions. Europeans were more likely to report either most of the time or always controlling fever (99/117; 85% vs 14/22; 64%; *P *= 0.034) and were more likely to use acetaminophen for fever control (82/117; 70% vs. 7/22; 32%; *P *= 0.001).

**Table 2:** Management practices for patients with fever admitted to intensive care units

Item	Never	Rarely	Sometimes	Most of the time	Always	*Median (IQR)
**Temperature is controlled in febrile patients**	0	3 (2%)	23 (17%)	57 (41%)	56 (40%)	4 (4 to 5)
**Temperature control in febrile patients is directed by physician order**	3 (2%)	18 (13%)	25 (18%)	54 (39%)	38 (28%)	4 (3 to 5)
**Temperature control in febrile patients is directed by nurses**	15 (11%)	41 (30%)	33 (24%)	28 (20%)	21 (15%)	3 (2 to 4)
**Acetaminophen (paracetamol) is used to control temperature in febrile patients**	2 (1%)	13 (9%)	35 (25%)	70 (50%)	19 (14%)	4 (3 to 4)
**Non-steroidal anti-inflammatory drugs (NSAIDs) are used to control temperature in febrile patients**	36 (26%)	48 (35%)	41 (30%)	13 (9%)	0	2 (1 to 3)
**Physical cooling methods are used to control temperature in febrile patients**	1 (1%)	18 (13%)	71 (51%)	36 (26%)	13 (9%)	3 (3 to 4)
**New fever triggers a full septic work-up**	1 (1%)	6 (4%)	25 (18%)	82 (59%)	25 (18%)	4 (4 to 4)
**Empiric antimicrobials are provided to febrile patients**	1 (1%)	24 (17%)	68 (49%)	39 (28%)	7 (5%)	3 (3 to 4)

*Median score (IQR, Interquartile range) of response with 1 = Never, 2 = Rarely, 3 = Sometimes, 4 = Most of the time, and 5 = Always

**Table 3:** Temperature control practices in selected patient diagnostic subgroups

Item	Never	Rarely	Sometimes	Most of the time	Always	*Median (IQR)
**Acute neurological injury (brain or spinal cord)**	1 (1%)	3 (2%)	7 (5%)	28 (20%)	100 (72%)	5 (4 to 5)
**Septic shock**	4 (3%)	8 (6%)	29 (21%)	45 (32%)	53 (38%)	4 (3 to 5)
**Severe sepsis without shock**	6 (4%)	13 (9%)	28 (20%)	50 (36%)	42 (30%)	4 (3 to 5)
**Infection without sepsis**	9 (6%)	21 (15%)	32 (23%)	42 (30%)	35 (25%)	4 (3 to 5)
**Acute liver failure**	9 (6%)	24 (17%)	38 (27%)	38 (27%)	30 (22%)	3 (3 to 4)
**Chronic liver failure**	11 (8%)	30 (22%)	47 (34%)	31 (22%)	20 (14%)	3 (2 to 4)
**Acute coronary syndrome**	9 (6%)	12 (9%)	38 (27%)	49 (35%)	31 (22%)	4 (3 to 4)

*Median score (Interquartile range, IQR) of response with 1 = Never, 2 = Rarely, 3 = Sometimes, 4 = Most of the time, and 5 = Always

An exploratory analysis was conducted by comparing survey results with clinical variables obtained in the original EUROBACT study. No significant relationships were found between reported temperature thresholds in the survey for defining fever and median time to adequate therapy, case-fatality rate or the proportion of patients with fever in the original study. In addition, participating ICUs were dichotomized into those that had case-fatality rates of 30% or less (low) and those that were greater than 30% (high) for hospital acquired bloodstream infection requiring ICU admission. No relationship between any of the survey variables and high or low case-fatality rate was observed.

## Discussion

This study documents major variability in reported practices in the diagnosis and management of temperature abnormalities in critically ill patients worldwide. A total of 14 different discrete thresholds for fever were reported in this study confirming that there is not widespread accepted levels or consensus for the diagnosis of fever. Furthermore, the modalities used to measure temperature varied widely across study centers (Table 1). Although a consensus panel representing the American College of Critical Care Medicine and the Infectious Diseases Society of America recommended use of a temperature of 38.3°C or higher to represent fever, the results of this survey indicate that this definition has not been widely adopted [3]. Adoption of standard definitions and methods of measurement are needed for studies defining the determinants and outcomes of temperature abnormalities in the critically ill.

Although a wide range of temperature measurement sites and devices were reportedly employed in study ICUs, it is important to note that these vary in their accuracy in determining true core temperature. While pulmonary artery catheters are widely accepted as a gold standard for temperature measurement in critically ill patients, the use of this device in ICUs has significantly decreased in recent years [21]. Relatively few studies have directly compared other measurement techniques against this gold standard in ICU patients [22]. Erickson *et al*. compared infrared tympanometry, bladder, oral and axillary temperatures with pulmonary artery thermistor measurements in 38 patients admitted to ICU [23]. They found generally good average measurement differences among the different techniques except that axillary thermometry significantly underestimated true temperature. In addition, the accuracy of each technique varied to different extents depending on the levels of pulmonary artery temperature. Lawson *et al*. evaluated temporal artery, tympanic membrane, oral and axillary thermometers against pulmonary artery catheter temperature among 60 adults with cardiopulmonary disease admitted to ICU [24]. They found that axillary measurement typically underestimated true temperature, that oral and temporal artery measurements were the most accurate, and that tympanic measurements were the least accurate and precise. Myny *et al*. evaluated axillary and temporal artery thermometry as compared to pulmonary artery catheter in 57 mostly normothermic adult patients admitted to ICU in Belgium [25]. They found good agreement between temporal artery thermometry and pulmonary artery catheter but that axillary thermometry was biased by approximately 0.5 degrees. In another study conducted in 14 critically ill patients, Stelfox and colleagues found that while the temporal artery thermometer had good concordance at normal temperatures as compared to bladder thermometry, it was highly inaccurate at extremes of temperatures [26].

While a range of responses was given, most respondents indicated that they treat fever in critically ill patients most or all of the time (Table 2). Though there is generally good theoretical and clinical evidence to support lowering of temperature in febrile patients with neurological compromise, this is not the case for most other critically ill patients. Five randomized clinical trials (RCTs) have been published that have assessed the effects of anti-pyretic therapy on neurologically intact febrile patients admitted to ICUs [9,11-14,27]. Four of these studies found no difference in outcome associated with temperature lowering therapy but were limited by small sample size [9,11-13]. On the other hand, Schulman *et al*. compared a strategy of aggressive versus permissive fever control in critically ill surgical patients and found that there was a trend toward increased mortality associated with fever treatment that necessitated stopping the trial due to safety concerns [12]. It is also important to recognize that the effect of fever control on outcomes may be dependent on whether the fever is due to an infectious etiology or not [5,6].

Despite the lack of evidence to support treatment of fever in most patients admitted to ICU, this appears to be common practice as evidenced by this study and other reports in the literature. One study from Canada found that among 100 critically ill adults without acute neurological injury, 79 received pharmacologic and/or physical anti-pyretic therapy during an episode of fever [28]. Similarly, another study conducted in Japan and Korea found that more than 50% of patients admitted to ICUs were treated with temperature lowering therapy [5]. Notably, they found a significantly increased risk for death associated with non-steroidal anti-inflammatory drug or acetaminophen therapy in patients with sepsis. Moreover, an online survey of members of the Australian and New Zealand Intensive Care Society Clinical Trials Group found that most respondents reported the use of interventions to lower temperature among febrile patients [29].

While this study provides insights into opinions and practices among a large number of centers, there are some important limitations that merit discussion. While EUROBACT does include representation from ICUs from six continents, most of the participants were European with disproportionate representation from France and Greece. Centers were not selected randomly from around the globe and as a result a significant selection bias is present. It is therefore not possible to accurately define different regional practices globally. A second limitation is that we reported responses in this study directly as entered by respondents without further validation or auditing of suspect data. As examples, one respondent reported using a remarkably high temperature of 40°C or higher to define fever and one reported that fever never triggered a septic workup (Table 2). Whether these are truly the cases or whether these are erroneous responses is not known. A third limitation is that the responses in this study were reported practices from one individual from an ICU. These may not be reflective of what is actually done in true practice as would be revealed by a chart audit.

## Conclusions

This study documents wide variability in approaches to diagnosis and management of temperature abnormalities among critically ill patients. Furthermore, despite being potentially harmful, most respondents report that they treat neurologically intact febrile patients admitted to ICU with temperature lowering interventions. This study provides support for further efforts to define and implement best practices for management of temperature abnormalities in the critically ill.

## Key messages

• Temperature abnormalities, including fever and hypothermia, are common among patients admitted to ICUs.

• There is major variability among ICUs in the methods of temperature measurement and for defining temperature abnormalities.

• Broad practice variation exists among ICUs for the further investigation and management of patients with temperature abnormalities.

• Given the commonality, impact on outcome and wide practice variation in diagnosis and management of temperature abnormalities in ICUs, further investigation and consensus are warranted to establish best practices

## Abbreviations

ICU: Intensive Care Unit; IQR: Inter-quartile range; RCTs: Randomized control trials.

## Competing interests

None of the authors have financial or non-financial competing interests that would influence the conduct or reporting of this study.

## Authors' contributions

DN and KL conceived and designed the study, collected data, conducted the primary analysis and drafted the manuscript. AT and AV contributed to study design, data collection and analysis. JR, DK, GD and J deW contributed to study design and collection of data. JFT participated in study conception and design, collection of data and analysis. All authors contributed to critical revision and approval of the final manuscript.

The EUROBACT Study Investigators (listed by country) are Jeffrey Lipman, Department of Intensive Care Medicine, Royal Brisbane and Women's Hospital, Brisbane, Australia; Anne Leditschke, Helen Rodgers, Canberra Hospital Intensive Care Unit, Canberra Hospital, Canberra, Australia; David Milliss, Thomas Gottlieb, Intensive Care Services, Concord Hospital, NSW, Australia; Stuart Baker, Brigit Roberts, ICU, Sir Charles Gairdner Hospital, Perth, Australia; Peter Krafft, Silvia Bernreiter, Intensiv 1b, Hospital Rudolfstiftung, Vienna, Austria; Pieter Depuydt, Intensieve Zorg, Universitair Ziekenhuis Gent, Ghent, Belgium; Philippe Jamaer, ICU A3 and C3, Jessa Hospital, Campus Virga Jesse, Hasselt, Belgium; Hervé Lebbinck, Iz Az Sint Augustinus Veurne, Veurne, Belgium; Frederico Bruzzi De Carvalho, Juliana Pereira, Centro De Terapia Intensiva, Hospital Mater Dei, Belo Horizonte, Brazil; Aline Camille Yehia, Felipe Carrhá Machado, Ana Luiza Horta de sa Carneiro, Cti Hospital, Julia Kubitschek, Belo Horizonte, Brazil; Antonio Fagundes Jr, Unidade De Terapia Intensiva, Hospital Do Coração Do Brasil, Brasília, Brazil; Fernando Rodriguez, Cti Geral, Hospital De Clínicas Niterói, Niterói, Brazil; Marcio Soares, Jorge Salluh, Cti, Instituto Nacional De Cancer, Rio de Janeiro, Brazil; Renata Beranger, ICU, São Lucas Hospital, Rio de Janeiro, Brazil; Marcelo Lugarinho, Cti Do Hospital De Clínicas Mario Lioni, Hospital De Clínicas Mario Lioni, Rio de Janeiro, Brazil; Alexandre Carvalho, Livia Reis, Uti 1-2-3, Udi Hospital, São Luis, Ma, Brazil; Cyntia de Lima, Uti Clínica, Hospital Santa Izabel, Salvador, Brazil; Claudio Piras, Cpc, Vitoria Apart Hospital, Vitoria, Brazil; Eliana Caser, Jansen Falcão, Uti Geral Adulto, Centro Integrado De Atençaõ A Saúde, Cias Unimed Vitória, Vitória, ES, Brazil; Kevin B. Laupland, ICU, Peter Lougheed Centre, Calgary, Canada; Kevin B. Laupland, ICU, Foothills Medical Centre, Calgary, AB, Canada; Kevin B. Laupland, CVICU, Foothills Medical Centre, Calgary, AB, Canada; Kevin B. Laupland, ICU, Rockyview General Hospital, Calgary, AB, Canada; Zhidan Zhang, Xiaochun Ma, Department of Critical Care Medicine, The First Affiliated Hospital of China Medical University, Shenyang, China; Xian Yao Wan, Jiu Zhi Zhang, Department of Intensive Care Medicine, The First Affiliated Hospital of Dalian Medical University, Dalian, China; Ke-Jian Qian, Liang Xia, Intensive Care Unit, The First Affiliated Hospital of Nan Chang University, Nan Chang, China; Congshan Yang, Department of Critical Care Medicine, Zhongda Hospital, Southeast University, Nanjing, China; Deng Lijing, Central ICU, West China Hospital of Sichuan University, Chengdu, China; Meili Duan, Department of Critical Care Medicine, Beijing Friendship Hospital, Capital Medical University, Beijing, China; Tang Zhanhong, Pan Yiping, Intensive Care Unit, The First Hospital of Guangxi Medical University, Nanning, China; Wang Yongqiang, Luo Ning, ICU, Tianjin First Center Hospital, Tianjin, China; Zhou Li-Xin, LI Jin-Quan, Intensive Care Unit, The Affiliated Foshan Hospital of Sun Yat-Sen University, Foshan, China; Xian Yao Wan, ICU, Beijing Tongren Hospital, Beijing, China; Ivan Gornik, Medical Intensive Care Unit, University Hospiral Centre Zagreb, Zagreb, Croatia; Vesna Degoricija, Medical ICU, University Hospital Sisters of Mercy and University of Zagreb School of Medicine, Zagreb, Croatia; Achille Kouatchet, Département De Réanimation Médicale Et Médecine Hy, Chu Angers, Angers Cedex 9, France; Gaetan Plantefeve, Olivier Pajot, Réanimation Polyvalente, Ch Victor Dupouy, Argenteuil, France; Hatem Kallel, Polyvalent ICU, Andrée Rosemon, Cayenne, France; Lherm Thierry, Kalfon Pierre, Réanimation Polyvalente, Louis Pasteur, Chartres, France; David Petitpas, Réanimation polyvalente, Chg Châlons En Champagne, Châlons En Champagne, France; Henry Lessire, Réanimation Médicale, Ch Pasteur, Colmar, France; Christian Brun-Buisson, Tai Pham, Réanimation Médicale, Chu Henri Mondor, Créteil, France; Djillali Annane, Virginie Maxime, Réanimation Médicale, Chu, Garches, France; Herault Marie-Christine, Reanimation Polyvalente Chirurgicale, Chu Michallon, Grenoble, France; Sybille Merceron, Medico-Surgical ICU, André Mignot Versailles Hospital Centre, Le Chesnay, France; Eric Kipnis, Marielle Boyer-Besseyre, Réanimation Chirurgicale, CHRU De Lille, Lille, France; Benoit Tavernier, Sebastien Faivre, Réanimation Neurochirurgicale, CHRU de Lille, Lille, France; Voillet Francois, Renaud Lepaul Ercole, Medical Intensive Care Unit, Hopital North, Marseille, France; Vincent Willems, Réanimation Polyvalente, Centre Hospitalier De Meaux, Meaux, France; Kada Klouche, Jean Philippe Delabre, Medical Intensive Care Unit, Lapeyronie University Hospital, Montpellier, France; Cartier Julien, Gleyse Brigitte, Service De Réanimation Polyvalente, Ch De Montélimar, Montélimar, France; Sebastien Gibot, Réanimation Médicale, Hopital Central, Nancy, France; Bruno Mégarbane, Réanimation Médicale Et Toxicologique, Hôpital Lariboisière, Paris, France; Philippe Seguin, Réanimation Chirurgicale, Chu De Rennes, Rennes, France; Anne Launoy, Service De Réanimation Chirurgicale Hautepierre, Hôpitaux Universitaires De Strasbourg, Strasbourg, France; Tixier Vincent, Medical, Gabriel Montpied, Clermont-Ferrand, France; Samir Jamali, Unité De Soins Intensifs, Centre Hospitalier De Dourdan, Dourdan, France; Silvia Calvino, Alexis Tabah, Réanimation Médicale, Grenoble Teaching Hospital, Grenoble, France; Michel Durand, Marine Rossi-Blancher, Reanimation Cardiovasculaire et Thoracique, Hopital Michallon, Grenoble, France; Alexandre Debrumetz, Elie Azoulay, Service De Réanimation Médicale, CHU Saint Louis, Paris, France; Julien Charpentier, Jean-Daniel Chiche, Réanimation Médicale Polyvalente, Cochin, Paris, France; Maité Garrouste-Orgeas, Benoit Misset, Réanimation Polyvalente, Paris Saint-Joseph, Paris, France; Gernot Marx, Klinik Für Operative Intensivmedizin Und Intermediate Care, University Hospital Aachen, Aachen, Germany; Wolfgang A. Krueger, Anaesthesiology and Intensive Care Medicine, Clinics of Constance, Constance, Germany; Thomas Felbinger, Department of Anaesthesiology, The Munich Municipal Hospitals Ltd., Munich, Germany; Alexandra Heininger, ICU 20-22, Universitätsklinik Für Anaesthesiologie Und Intensivmedizin, Tuebingen, Germany; Ingo Voigt, Kardiologische Intensivstation, Elisabeth Krankenhaus, Essen, Germany; Torsten Schroeder, Interdisziplinäre Intensivstation, Karl-Olga Krankenhaus, Stuttgart, Germany; Ioannis Pneumatikos, Vassiliki Theodorou, Critical Care Department, University Hospital of Alexandroupoli, Alexandroupoli, Greece; Despoina Koulenti, Apostolos Armaganidis, 2^nd ^Critical Care Department, Attikon University Hospital, Athens, Greece; Pavlos Myrianthefs, Alexandra Gavala, Athens University, School of Nursing, ICU, "Kat" General Hospital, Athens, Greece; Chara Nikolaou, Katerina Kounougeri, Department of Critical Care Medicine, Konstantopouleion General Hospital of Nea Ionia, Athens, Greece; Christina Routsi, Adamantia Liapikou, University ICU Department, Evangelismos General Hospital, Athens, Greece; Christodoulos Nathanail, Pirros Tsakas, Intensive Care Unit, General Hospital of Arta, Arta, Greece; Andreas Karabinis, Christos Tsakalakis, Critical Care Department, General Hospital "G. Genimatas," Athens, Greece; Kostas Mandragos, Chrysostomos Katsenos, Intensive Care Unit, Red Cross (Erythros Stavros) Hospital, Athens, Greece; Georgios Anthopoulos, Georgios Choutas, Intensive Care Unit, 251 Air Force General Hospital, Athens, Greece; Anastasia Koutsikou, Ilona Nikolaidou, Intensive Care Unit (Surgical), General Hospital of Athens "Asklepieion Voulas," Athens, Greece; Vasileios Bekos, Anna Spring, Intensive Care Unit, Naval Hospital of Athens, Athens, Greece; Haralambos Paskalis, Vassiliki Psallida, Intensive Care Unit, Hygeia General Hospital, Athens, Greece; Aikaterini Ioakeimidou, Alexandra Lahana, Intensive Care Unit, Athens Veterans Hospital (Nimits), Athens, Greece; Paraskevi Plantza, Aikaterini Nodarou, ICU Kaa Sotiria General Hospital, Sotiria General Hospital, Athens, Greece; Antonia Koutsoukou, Magdalini Kyriakopoulou, ICU 1st Resp. Medicine Depart. Athens University, Sotiria General Hospital, Athens, Greece; Martha Michalia, Phyllis Clouva-Molyvdas, Department of Critical Care Medicine, "Thriassion" General Hospital of Eleusis, Elefsina, Athens, Greece; Dimitrios Sfyras, Christos Georgiadis, Intensive Care Unit, General Hospital of Lamia, Lamia, Greece; Pavlos Polakis, Spiros Papanikolaou, Intensive Care Unit, "Peiraiko" Therapeftirio, Pireus, Greece; Christos Christopoulos, Efstratia Vrettou, Intensive Care Unit, General Hospital of Pyrgos, Pyrgos, Greece; Kostoula Arvaniti, Dimitrios Matamis, Critical Care Department, "Papageorgiou" General Hospital of Thessaloniki, Thessaloniki, Greece; Theoniki Paraforou, Kyriaki Spiropoulou, ICU, General Hospital of Trikala, Trikala, Greece; Dimitris Georgopoulos, Maria Klimathianaki, Critical Care Department, University General Hospital of Heraklion, Crete, Heraklion, Greece; Georgios Nakos, Vasilios Koulouras, Critical Care Department, University Hospital of Ioannina, Ioannina, Greece; Apostolos Komnos, Achilleas Chovas, Department of Critical Care Medicine, General Hospital of Larisa, Larisa, Greece; Athanasios Prekates, Eleni Magira, Critical Care Department, "Tzaneion" General Hospital of Pireus, Pireus, Greece; Maria Giannakoy, Eleni Gkeka, Intensive Care Unit, "Ahepa" General Hospital, Thessaloniki, Greece; Eleni Antoniadou, Elli Antypa, Critical Care Department, General Hospital of Thessaloniki "G. Genimatas," Thessaloniki, Greece; Nikoletta Gritsi-Gerogianni, Christina Kydona, Critical Care Department, "Hippokrateion" General Hospital of Thessaloniki, Thessaloniki, Greece; Epaminondas Zakynthinos, Nikolas Tzovaras, Critical Care Department, University Hospital of Larissa, Larissa, Greece; Akos Csomos, Surgical Intensive Care, Semmelweis University, Budapest, Hungary; Csóka Gábor, Sürgősségi Beteg Ellátó Egység, Fővárosi Önkormányzat Szent Imre Kórház, Budapest, Hungary; Borbala Mikos, György Velkey, Pediatric Anaesthesia and Intensive Care Unit, Bethesda Children's Hospital of The Hungarian Reformed Church, Budapest, Hungary; Eszter Vitális, Auguszta Sebészet Intenzív, The Medical And Health Science Centre of the University of Debrecen, Debrecen, Hungary; Nóra, Ágota Kovács, Aito Flór Ferenc, Kistarcsa, Hungary; Lajos Bogar, Tamas Kiss, Department of Anaesthesia and Intensive Therapy, University of Pécs, Pécs, Hungary; Zollei Eva, Medical ICU, University of Szeged, Szeged, Hungary; Valerio Mangani, Giorgio Tulli, Intensive Care Unit, S. Giovanni Di Dio, Firenze, Italy; Guido Stefania, Ronco Chiara, Centro Rianimazione, Maggiore Della Carità, Novara, Italy; Massimo Antonelli, Luca Montini, Rianimazione E Terapia Intensiva, Policlinico Universitario A. Gemelli, Rome, Italy; Monica Rocco, Giorgia Citterio, Centro Di Rianimazione, Policlinico Umberto I, Rome, Italy; Shigeki Fujitani, Emergency & Critical Care Medicine, St. Marianna University School of Medicine Hospital, Kanagawa, Japan; Koji Hosokawa, Intensive Care Unit, Kyoto Prefectural University of Medicine, Kyoto, Japan; Motaouakkil Said, Charra Boubaker, Reanimation Medicale, CHU Ibn Rochd, Casablanca, Morocco; Marcus Schultz, Annelou Van Der Veen, ICU, Academic Medical Center, Amsterdam, The Netherlands; Heleen Aardema, Intensive and Respiratory Care Unit, University Medical Center Groningen, Groningen, The Netherlands; Dennis Bergmans, Rik Schoemakers, Department of Intensive Care, Maastricht University Medical Centre, Maastricht, The Netherlands; Ronald Wesselink, ICU, St. Antonius Ziekenhuis, Nieuwegein, The Netherlands; Evelien A.N. Oostdijk, Marc J.M. Bonten, Department of Medical Microbiology, University Medical Center Utrecht, Utrecht, The Netherlands; Iwona Dragan, ICU, General Hospital, Gniezno, Poland; Włodzimierz Kostyrka, Medical ICU, Szpital Powiatowy, Ostrów Wielkopolski, Poland; Barbara Tamowicz, Adam Mikstacki, Department of Anaesthesiology and Intensive Therapy, Poznan University of Medical Sciences, Regional Hospital, Poznan, Poland; Piotr Smuszkiewicz, Department of Anaesthesiology and Intensive Therapy, University Hospital, Poznan, Poland; Jacek Nadolski, Oa I It, Wielkopolska Center of Pulmonology and Thoracic Surgery, Poznań, Poland; Robert Choma, Oddział Anestezjologii I Intensywnej Terapii, Szpital W Śremie, Śrem, Poland; Wladyslaw Koscielniak, Pawel Pietraszek, Oddzial Anestezjologii I Intesywnej Terapii, Regional Hospital Zielona Gora, Zielona Gora, Poland; Edward Maul, Serviço De Medicina Intensiva, Hospital Central Do Funchal, Funchal, Portugal; Anabela Bártolo, Salomé Martins, UCIP, Chaa, Guimarães, Guimarães, Portugal; Isabel Miranda, Mariana Oliveira, UCIP02, Hospital De St. António Dos Capuchos, Centro Hospitalar De Lisboa Central, E.P.E., Lisboa, Portugal; Carlos França, Ana Tornada, Smi, Hospital De Santa Maria, Lisbon, Portugal; Luís Telo, Leonardo Ferreira, UCIP, Pulido Valente, Lisboa, Portugal; Teresa Cardoso, Unidade De Cuidados Intensivos Polivalente, Hospital De Santo António, Porto, Portugal; Lurdes Santos, Alcina Ferreira, UCI-DI, Hospital S. João, Porto, Portugal; José Manuel Pereira, UCIP Geral, Hospital S João, Porto, Portugal; Celeste Dias, UCI Neurocriticos, Hospital Sao Joao, Porto, Portugal; Maria Conceição Dias, UCIPU, UCIP Urgencia, Hospital De S. João, Porto, Portugal; Ana J. Marques, Paula Castelões, Ucipolivalente Do Chvngaia, Hospital Santos Silva, Centro Hospitalar Vila Nova De Gaia, Vila Nova Gaia, Portugal; Uros Batranovic, Srdjan Gavrilovic, Intensive Care Unit, Institute For Pulmonary Diseases of Vojvodina, Sremska Kamenica, Republic of Serbia; Daniela Filipescu, Cardiac Anesthesia and Intensive Care, Emergency Institute of Cardiovascular Diseases, Bucharest, Romania; Francisco Alvarez-Lerma, Maria Pilar Gracia, Intensive Care Unit, Hospital Del Mar, Barcelona, Spain; Fernando Armestar-Rodriguez, Eduard, Mesalles-Sanjuán, Medicina Intensiva, Hospital Universitari Germans Trias I Pujol, Badalona, Spain; Nerea Lopez De Arbina, Josep Sirvent, Servicio De Medicina Intensiva, Hospital Universitari De Girona Dr Josep Trueta, Girona, Spain; Pau Garro, UCI General, Hospital General De Granollers, Granollers (Barcelona), Spain; Juan Ramón Cortés Cañones, Unidad Cuidados Intensivos, Complexo Hospitalario Ourense Cristal Piñor, Orense, Spain; Armando Blanco, Lara Marqués, Unidad De Medicna Intensiva I, Hospital Universitario Central De Asturias (HUCA), Oviedo, Spain; Josu Insausti, Iñigo Martija, UCI, Hospital De Navarra, Pamplona, Spain; Jordi Valles, Ricard Ferrer, Critical Care Center, Hospital Sabadell, Sabadell, Spain; Alejandro Ubeda, Francisco Lucena, Polyvalent ICU, H.U. Valme, Seville, Spain; Maricarmen Gilavert Cuevas, UCI, Hospital Universitario Joan Xxiii-Instituto Pere Virgili, Tarragona, Spain; Rafael Zaragoza, Susana Sancho, ICU, Hospital Univ. Dr. Peset, Hospital Universitario Dr. Peset, Valencia, Spain. Markus Laube, Madeleine Rothen, Intensivstation, Spitalzentrum, Biel, Switzerland; Philippe Eggimann, Jean-Luc Pagani, Service De Médecine Intensive Adulte, Chuv, Lausanne, Switzerland; Samia Ayed, Service De Reanimation Medicale, CHU Tahar Sfar, Mahdia, Tunisia; Islem Ouanes, Fekri Abroug, Réanimation Polyvalente, Chu Fattouma Bourguiba, Monastir, Tunisia; Dilek Özcengiz, Reanimation, Cukurova Medical University, Adana, Turkey; Seyhan Yağar, Cardiovascular Surgery, ICU, Türkiye Yüksek Ihtisas Hospital, Ankara, Turkey; Süheyla Ünver, Yeliz Irem Tunçel, Anestesia Intensive Care Unit, Ankara Dr Abdurrahman Yurtaslan Onkoloji E. A. Hastanesi, Ankara, Turkey; Unase Buyukkocak, Esra Aykac, Intensive Care Unit and Anaesthesia, Kirikkale University, The School of Medicine Hospital, Kirikkale, Turkey; Ahmet COŞAR, Hüseyin Oğuz Yilmaz, Anesteziyoloji Ve Reanimasyon Ad Ybü, Gülhane Askeri Tıp Akademisi, Ankara, Turkey; Arash Pirat, Pinar Zeyneloglu, Surgical Intensive Care Unit, Baskent University Hospital, Ankara, Turkey; Nermin Kelebek Girgin, Halis Akalın, Anaesthesiology and ICU, Uludag University Medical Faculty, Bursa, Turkey; Hulya Sungurtekin, Simay Serin, Anaesthesiology and ICU, Pamukkale University, Denizli, Turkey; I. Ozkan Akinci, Neuro Icu, Istanbul Medical Faculty, Istanbul, Turkey; Tayfun Adanir, Atilla Sencan, Anaesthesiology and ICU, Ataturk Training And Research Hospital, Izmir, Turkey; Ahmet Dilek, Mikail Yüksel Intensive Care Unit, Ondokuz Mayis University, School of Medicine, Samsun, Turkey; Ismail KATI, Ugur Goktas, Anaesthesia and Intensive Care Unit, Yuzuncu Yil University Medical Faculty, Van, Turkey; Ashraf El Houfi, MICU-SICU, Dubai Hospital, Dubai, United Arab Emirates.

## Supplementary Material

Additional file 1 **EUROBACT Investigators Survey**. This survey was sent to study participants. Click here for file
