# Supplementary material for: Diagnosis and management of temperature abnormality in ICUs: a EUROBACT investigators' survey
Source: Crit Care. 2013 Dec 10;17(6):R289. doi: 10.1186/cc13153 (PMC4057370; doi:10.1186/cc13153)
Supplement: Additional file 1 — EUROBACT Investigators Survey. This survey was sent to study participants. [file cc13153-S1.DOCX]

**EUROBACT Investigators Survey**

***Please complete the following questionnaire on behalf of your ICU. Individual intensivists may practice differently at your centre. Please respond from the perspective of the usual or average practice in your ICU.***

1. Is there a specific temperature threshold that defines fever in your ICU?

[ ] Yes

[ ] No

1. If the answer to question #1 is yes, what is the temperature threshold?

**≥** |__|__|.|__|^O^C

1. Is the use of thermometers protocolized in your ICU? [ ] Yes [ ] No
2. From the following list of thermometers, select which are currently employed in your ICU and provide the order of frequency of their use.

| **Thermometer** | **Used to Measure Temperature in Your ICU?** | **List Order of Frequency of Use (ie 1=most common, 2=second most common, etc)** |
| --- | --- | --- |
| Tympanic membrane | [ ] Yes [ ] No | ____________ |
| Temporal artery | [ ] Yes [ ] No | ____________ |
| Axillary | [ ] Yes [ ] No | ____________ |
| Oral | [ ] Yes [ ] No | ____________ |
| Rectal | [ ] Yes [ ] No | ____________ |
| Esophageal | [ ] Yes [ ] No | ____________ |
| Urinary bladder | [ ] Yes [ ] No | ____________ |
| Pulmonary artery catheter | [ ] Yes [ ] No | ____________ |
| PICCO | [ ] Yes [ ] No | ____________ |
| Other: _______________ | [ ] Yes [ ] No | ____________ |

1. In the event of new fever, which of the following best describes the approach in your ICU with respect to the ordering of blood cultures (circle one only)?
   1. Routinely initiated by nurses (as protocolized) if the temperature reaches a threshold ?
   2. Only if prescribed by specific physician order
   3. Routinely performed by nurses unless physician order not to do
   4. Other : please describe : ________________________________________
2. In case of hypothermia, what is your usual threshold to perform blood culture? ≤ |__|__|.|__|^O^C
   1. Routinely initiated by nurses (as protocolized) if the temperature reaches a threshold ?
   2. Only if prescribed by specific physician order
   3. Routinely performed by nurses unless physician order not to do
   4. Other : please describe : ________________________________________
3. When blood cultures are ordered in response to new fever do you

- 1. Systematically use bottles for aerobic culture? [ ] Yes [ ] No
  2. Systematically use bottles for anaerobic culture? [ ] Yes [ ] No
  3. Systematically use bottles for fungi? [ ] Yes [ ] No
  4. Specifically specify which bottles to use by physician order? [ ] Yes [ ] No

1. Is there a formal written protocol in place for temperature control among febrile patients in your ICU?

[ ] Yes

[ ] No

1. With regard to fever management in your ICU, please evaluate the following statements using to the 5-point scale below

|  | Never (1) | Rarely (2) | Sometimes (3) | Most of the time (4) | Always (5) |
| --- | --- | --- | --- | --- | --- |
| 1- Temperature is controlled in febrile patients | [ ] | [ ] | [ ] | [ ] | [ ] |
| 2- Temperature control in febrile patients is directed by physician order | [ ] | [ ] | [ ] | [ ] | [ ] |
| 3 -Temperature control in febrile patients is directed by nurses | [ ] | [ ] | [ ] | [ ] | [ ] |
| 4 - Acetaminophen (paracetamol) is used to control temperature in febrile patients | [ ] | [ ] | [ ] | [ ] | [ ] |
| 5 -Non-steroidal anti-inflammatory drugs (NSAIDs) are used to control temperature in febrile patients | [ ] | [ ] | [ ] | [ ] | [ ] |
| 6 - Physical cooling methods are used to control temperature in febrile patients | [ ] | [ ] | [ ] | [ ] | [ ] |
| 7 - New fever triggers a full septic work-up | [ ] | [ ] | [ ] | [ ] | [ ] |
| 8 - Empiric antimicrobials are provided to febrile patients | [ ] | [ ] | [ ] | [ ] | [ ] |
| Temperature is controlled in the following patient diagnostic subgroups |  |  |  |  |  |
| 10 -Acute neurological injury (brain or spinal cord) | [ ] | [ ] | [ ] | [ ] | [ ] |
| 11 -Septic shock | [ ] | [ ] | [ ] | [ ] | [ ] |
| 12-Severe sepsis without shock | [ ] | [ ] | [ ] | [ ] | [ ] |
| 13- Infection without sepsis | [ ] | [ ] | [ ] | [ ] | [ ] |
| 14 -Acute liver failure | [ ] | [ ] | [ ] | [ ] | [ ] |
| 15 -Chronic liver failure | [ ] | [ ] | [ ] | [ ] | [ ] |
| 16 - Acute coronary syndrome | [ ] | [ ] | [ ] | [ ] | [ ] |
